# Composite Pre-heating: A Novel Approach in Restorative Dentistry

**DOI:** 10.7759/cureus.27151

**Published:** 2022-07-22

**Authors:** Jay Bhopatkar, Anuja Ikhar, Manoj Chandak, Nikhil Mankar, Shweta Sedani

**Affiliations:** 1 Conservative Dentistry and Endodontics, Sharad Pawar Dental College And Hospital, Datta Meghe Institute Of Medical Sciences University, Wardha, IND

**Keywords:** polymerization shrinkage, microleakage, pre-heating, composite pre-heating, composite resins

## Abstract

Resin composite pre-heating is a novel approach that might improve handling and marginal adaptation of the unset material paste in clinical application. The goal of this review article is to compile all laboratory experiments on resin composite preheating and see how it impacts the mechanical properties of the material. Results have shown that preheating composite resins improves the degree of conversion, stiffness, marginal adaptability, and microhardness. While flexural strength is unbothered, polymerization shrinkage is hindered, and the microleakage results are unknown.

## Introduction and background

During the last decade, the application of direct resin composites in general clinical practice has increased due to the growing patient demand for cosmetic restorations [1]. Composite resins have superior physical and chemical qualities, great operability, and good aesthetic performance when compared to conventional repair materials like silver amalgam. However, the polymerization reaction during the curing process is always followed by varying degrees of volume shrinkage in the composite resins with bis methacrylate groups that are frequently utilized in clinical practice (2.7-7.1%) [2]. The stress that follows could result in bond failure, along with some other unfavorable clinical outcomes like microcracks in the repair material, enamel fracture, and a gap between the composite resin and the nest wall that could cause secondary caries and postoperative sensitivity, all of which could have an impact on the effectiveness of the long-term filling repair. Composites, on the other hand, have drawbacks such as stickiness and great viscosity, which make them tricky to handle and manipulate. As a result, marginal adaptability is minimal to prepared walls [3-5]. Due to their better flow capacity, flowable composites may be able to reduce the gaps around the prepared wall and the restoration. They may, however, undergo higher net shrinkage due to the reduced filler content, jeopardizing the repair's mechanical characteristics. Another option is to utilize a flowable liner in combination with standard composites to solve the problem. Although using traditional composites which have been warmed in an external warming device prior to polymerization is a novel concept [6-9].

In clinical practice, photocuring resin, a composite material that contains a light-curing initiator system made up of photosensitizers and accelerators, is frequently utilized. The photosensitizer breaks down the light-cure composite resin's polymerization reaction into free radicals under irradiation at a specific wavelength. The free radicals then act with the monomer to cause it to form new free radicals, which then start a chain reaction that causes the resin matrix and diluent to polymerize and cure. Recently, studies have discovered that preheating resins can boost conversion by reducing resin viscosity, accelerating free radical motion, and increasing the frequency at which unreactive groups and free radicals collide [10,11]. Both the rate of conversion of composite resin monomers and their mechanical properties increase. The decrease in viscosity is an effect of heat energy that separates monomers and oligomers, making it simpler for them to glide by one another [1,3]. The enhanced outflow of pre-warmed composite would, in principle, enhance uncured resin adaptation to prepared walls and perhaps minimize leakage [12]. The film thickness is greatly reduced, and the fluidity is increased after the composite resin is heated. This can improve the material's adaptation to the cavity and facilitate the sealing between the resin and the tooth tissue. However, while preheating, resins can improve edge adaptability and monomer conversion, it can also increase polymerization shrinkage, which can negatively impact microleakage [6]. Therefore, research into how preheating affect composite resins is crucial for its clinical use. Potential benefits of composite preheating include greater marginal adaptation, higher monomer conversion, and increased mechanical characteristics. As a result of these factors, it has become a popular treatment option among dentists [8,9,12,13].

This report's objective is to assess the material and advantages of resin composite preheating by compiling the results of investigations that examined the practice.

## Review

Preheating of composite resins

Composite resins are nowadays commonly employed as restorative materials and as a mercury (Hg)-free alternative to silver amalgam due to their poor mechanical and aesthetic properties [14]. Although composite resins have shortcomings such as polymerization shrinkage, post-restoration sensitivity, poor proximal contact, limited wear tolerance, and insufficient confirmation in some therapeutic circumstances, they do have certain advantages [15]. Friedman was the first to implement preheating in 2001 [16]. The advantages of pre-warming used to upscale the mechanical characteristics of composite resins include improved restoration longevity and stress reduction [15,17], superior adaptability [18], and faster curing time [19]. However, Daronch et al. found no significant variation between composite resins that had been warmed and composite resins that had not been preheated [20]. The drawbacks of composite preheating are that it reduces shelf life and necessitates speedy operations [21]. Pulpal irritability is a question when composite resins are heated to 55-69°C. Despite this, experiments show that placing a 60°C warmed composite resin raises pulp temperature by just 0.8°C, whereas 15 seconds of light-curing raises pulp temp by 4.5-5°C [22]. According to Daronch et al., after withdrawing composites from the external warming machine, 50% of the temperature obtained is lost in 120 seconds, and about 90% did lose in 300 seconds [20].

Curing mechanism of preheated composite resins

A highly cross-linked network with covalent linkages between the polymer chains is created during the composite resin's polymerization phase. After photopolymerization, the crosslinking density and conversion rate grow quickly, generating an infinite network, which causes a rapid increase in system viscosity. This first transition, known as gelation, occurs when viscous liquids turn into elastic gels [23]. Free radicals on large molecules (growth polymer chains) are more affected by mobility limitations at the gel point than free radicals on small monomer molecules, which can still diffuse readily. As a result, bimolecular termination is considerably diminished, yet initiation can still produce new polymerization extension centers. As a result, an increase in free radical concentration causes a sudden rise in the rate of polymerization known as auto-acceleration [24].

Viscosity increases during the course of the reaction, restricting even the diffusion of monomer molecules and significantly reducing auto-acceleration. This reflects the transition from a second state, gelatinous, to vitrification [25]. This explains why, even under ideal irradiation circumstances, the monomer conversion rate cannot approach 100%. Vitrification blocks any additional substantial polymerization activities. The increase in temperature during the preheating process causes the glassing process to be delayed. Because the material can still be further polymerized until the glass transition temperature reaches the polymerization reaction temperature, higher curing temperatures result in a greater final monomer conversion rate [26].

Effect of preheating on various properties of composite resins

Effect of Preheating on the Color of Composite Resin

The following elements influence the composite resin's color: 1) its internal light scattering and light absorption properties, 2) its thickness, and 3) the backdrop materials' light reflection properties. The hue of the composite resin might change for a variety of causes, including inherent or exogenous variables.

The Tetric N-Ceram nanocomposite resin conversion rate was tested by Mundim et al. [27], and the findings revealed that while the conversion rate was improved, preheating had little impact on the composite resin's optical characteristics. According to the Kahnamouei et al. investigation, it was not obvious whether there was a difference between 20 and 40 preheating cycles when evaluating color changes and detecting significant variations [28]. The research findings demonstrate that repeated preheating cycles have a detrimental effect on the color stability of the composite resin, and it is advised to refrain from heating the resin repeatedly.

Effect of Preheating on the Conversion Rate of Composite Resin Monomers

When photoinitiated bis-methacrylate-based materials polymerize, the double bond transformation is insufficient, leaving a sizable part of the methacrylate groups unreacted [29]. This reaction is self-limiting because as it proceeds, the resin becomes more viscous and forms a strongly cross-linked polymer network, which causes radical mobility to dramatically decrease. The mechanical characteristics and biological safety of the composite resin are directly influenced by the non-polymerized residual monomer; the more residual monomers present, the lower the composite resin's mechanical strength and the more irritated the pulp is [30,31].

According to Daronch et al., preheating the composite resin prior to light curing can greatly enhance the conversion of the resin sample's top and bottom surfaces [12]. It was also discovered that preheated composite resin can accomplish equal conversion with a shorter exposure time, i.e., lower the exposure by 50-75% and yet get the same or higher conversion rate. Reduced exposure results in fewer free radicals being produced, but higher temperatures decrease the viscosity of the composite resin and increase radical mobility, which leads to more polymerization processes and higher conversion rates [32,33].

Preheated composite resins may induce a quick temperature fall during the transfer of access, limiting the effect, according to in vitro investigations [25]. However, there is still a larger conversion rate when compared to the effect obtained by the composite resin while it is at room temperature, even if the resin is preheated to 54 °C and quickly cooled (which may drop to 30 °C or 40 °C). The investigation discovered that a composite resin with an exposure duration of 10 s at a temperature of 40 °C can also achieve a greater conversion rate than a composite resin with an exposure time of 20 seconds at a temperature of 22 °C. Regardless of the light source or composite resin material employed, light-curing at a modest preheating temperature of 54.5 °C has a better immediate and ultimate conversion rate than light-curing at ambient temperature [34].

Effect of Preheating on the Fluidity of Composite Resin (Film-Forming Thickness)

According to Rueggeburg et al., the film-forming thickness was reduced by 30% when the resin was heated to 54 °C, demonstrating the reduced viscosity and improved fluidity of visco-elastic materials like composite resins at high temperatures. At room temperature, the film thickness of traditional composite resins varies. When employing common composite resins, certain warmed composites, but not all of them, will cause a decrease in the thickness of the film that forms. The preheated traditional composite resin cannot have the same low film-forming thickness value as the flowing resin at room temperature within the heating unit's temperature range. Therefore, flow resin applications cannot be replaced by preheated conventional composite resins.

Experiments have shown that heating significantly reduces the film-forming thickness of other goods in addition to SureFil® (Dentsply Sirona, Charlotte, North Carolina, United States) and Filtek™ A110 (The 3M Company, Saint Paul, Minnesota, United States) resins, demonstrating that each material's film-forming thickness decreases between 54 °C and 60 °C in relation to its room temperature [35]. Based on these findings, da Costa et al. hypothesize that when composites are heated clinically, their fluidity rises, and their film-forming thickness reduces [35].

Effect of Preheating on Microleakage at the Edge of Composite Resin Filler

The primary reason for microleakage at the margins is the polymeric shrinkage of the composite resin. After the composite resin is light-cured, the adhesive layer forms a solid bond, but as the composite resin polymerizes and shrinks, it may cause the adhesive to separate from the surface of the tooth tissue, especially if it destroys the adhesive layer between dentin and the adhesive. This will result in gap expansion at the interface between composite resin and tooth tissue. After a filling, microleakage of the edges can make teeth more sensitive, change the color of the edges, and possibly lead to secondary caries and loose shedding of restorations. Tantbirojn et al. discovered that the stress caused by the composite resin's polymerization contraction can deform the tooth tissue or restoration, leading to adhesion failure and significantly reducing the resin filling repair's long-term effectiveness [36].

Important factors influencing the creation of gaps around resin restorations include polymer shrinkage and the fluidity of composite resins. In order to better adhere to the cavity wall, flowable composite resins, as opposed to commonly used composite resins, reduce their viscosity by lowering the filler content and altering the chemical composition of the matrix. However, the polymerization shrinkage rate of the flowing composite resin is also higher. Currently, composite resin, which is frequently used in clinical practice, increases the filler content, improves the mechanical characteristics, and makes materials more robust, but doing so also makes the substance itself more viscous, less fluid, and less capable of sealing edges.

Preheating increases the fluidity of composite resins, according to studies [33,37]. By preheating the composite resin, Blalock et al. discovered that the viscosity, flowability, and microleakage of the composite resin may all be improved [38]. Theoretically, thermal vibration causes the resin monomers or oligomers to split, making them simpler to slide between one another, which results in composite resins having reduced viscosity. The fluidity of the composite resin is improved by preheating it. It also becomes more adaptable to the cavity wall and has better edge closure, which reduces microleakage. According to a study, preheated composite resins were also more effective at reducing microleakage from the neck edges of the teeth [7].

The monomer conversion increases noticeably with rising temperature when the composite resin is warmed prior to light curing. However, the viscosity flow and molecular fluidity in highly cross-linked polymers are decreased and the shrinkage stress is elevated as a result of the higher degree of transformation. The filler's interface may become stressed due to the heat produced during polymerization and the mismatch in the polymer matrix's coefficient of thermal expansion, which results in an internal "circumferential" stress surrounding the filler [10]. As temperature rises, residual tension also rises sharply.

Effect of Preheating on Stress and Curing Depth of Light-Cured Composite Resin

According to the study, preheating typical composite resins to 40 °C can reduce the light-curing time by half compared to room temperature (22 °C) without changing the properties of their hardness. Preheating the composite resin can thereby clinically boost the material's cure depth and cut the curing time in half [39]. Studies of isothermal polymerization conducted in vitro have revealed that the degree of conversion, polymerization rate, and maximal conversion rate all improve when composite curing takes place above room temperature [12]. However, research demonstrates that raising the polymerization conversion rate also raises the risk of polymerization stress development [40,41]. The maximum stress values are much higher than those of the composite resin material at room temperature under non-isothermal conditions when the composite resin is initially preheated at 60 °C. The viscosity of the system falls as the temperature of the composite resin is raised before the polymerization reaction, which leads to more monomer transformation and, consequently, higher polymerization stress.

Effect of Preheating on Wear Resistance, Microhardness, and Fracture Resistance of the Composite Resin

The proportion of the residual “Carbon to Carbon” double bonds is completely set resin composite to the total number of “Carbon to Carbon” double bonds in the unset material is called the degree of conversion [21,22]. The degree of conversion is a criterion for determining the mechanical qualities of restorative materials at the end of the process [22]. To put it another way, a lower degree of conversion reduces the microhardness, wear tolerance, fracture resistance, and flexural strength of composite resins [5,22,42]. Preheating composite resins has been proposed as a method of increasing the degree of conversion and, as a result, microhardness, wear tolerance, and fracture resistance [38,43-48]. As the temperature rises, the monomers become more mobile and the polymerized networks become more cross-linked [14,22]. Didron et al., on the other hand, discovered that preheating had zero influence on microhardness [49]. Eman et al., Lucy et al., Kashi et al., and Torres et al. all encourage the notion of preheating resin composites to improve mechanical characteristics [11,50-52]. The degree of conversion varies depending on the type and color tone of composite resin used, which explains the discrepancies in the above-mentioned studies [16].

Effect of Preheating on Flexural Strength of Composite Resins

The degree of conversion is correlated with flexural strength, which is a significant metric for fragile materials [16-19]. Although preheating composite resins enhances the degree of conversion [16], Uctasli et al., Nada et al., and Salgado et al. found that it had negligible influence on flexural strength. There is no discernible change in flexural strength between heated and unheated composites [4,9,16]. According to Amario et al., significantly repeated cycles of preheating can reduce the flexural strength of resin composites [18,53-55].

Effect of Preheating on the Viscoelasticity of Composite Resins.

Mesquita et al. discovered that the viscoelasticity of composite resins changes as test temperature rises, and this has a lot to do with the composition and structure of the resins' chemical makeup, as well as their levels of free monomers, water adsorption, and degree of curing [56]. No phase transition was noticed in the oral temperature range despite a drop in the elasticity modulus and an increase in the tangent of the viscosity modulus and loss angle. As a result, in their clinical applications, composite resins do not experience abrupt changes in their mechanical properties.

These results indicate that within the first few hours and days, light-cured dental composite resin restorations will still retain some uncured material. The quantity of non-responsive monomers will eventually decline due to the consumption of hot foods and beverages, normal oral temperature, and increased temperature induction. In other words, at room temperature, the composite resin will fully cure. These findings have clinical significance because they show that dental resin will continue to harden after polymerization at initial light and oral temperature. This will have both a positive and a negative effect on the composite resin's ability to repair the damage, such as increased rigidity and microleakage.

Effect of Preheated Composite Resin on Pulp.

The temperature of the pulp will alter as the composite resin is warmed and inserted into the tooth's cavity. The pulp can withstand high temperatures, according to studies [57]; however, it is not clinically advised to heat the composite resin above 60 °C since the pulp may suffer irreparable damage if the temperature climbs by 5.5 °C.

According to Freedman et al., the temperature of the pulp increased by only 1.6 °C when a 54.5 °C composite resin was injected into a tooth with a 1 mm remnant of dentin [58]. According to Rueggeberg et al., the temperature inside the pulp rose by an average of 7.9 °C when the composite resin was heated to 60 °C [59]. Other in vitro studies have demonstrated that using high-intensity light-curing lamps for longer periods of time increases the risk of pulp injury [60,61]. Additionally, in clinical settings, when the composite resin material is preheated, its temperature may affect the temperature of the pulp tissue. However, because of the delay between the composite resin's extrusion from the syringe tube and its insertion into the tooth cavity, its temperature may drop significantly [62]. Incomparable repair stages, Daronch et al. discovered no appreciable variation in pulp temperature between composite resin materials at room temperature and those that had been preheated [20]. Therefore, it is advised not to preheat the composite resin above 60 °C in order to avoid irritating the pulp.

The results obtained by various authors mentioned in this review describing the effect of preheating on mechanical properties of composite resins are summarized in Table 1.

**Table 1:** The laboratory tests and their results included in this study I: Increase; D: Decrease; N: No statistical difference * Under certain circumstances

STUDY	DEGREE OF MONOMER CONVERSION	MARGINAL ADAPTATION	FLEXURAL STRENGTH	VISCOSITY	MICRO-HARDNESS	MICRO-LEAKAGE	POLYMERIZATION SHRINKAGE
Daronch et al., 2005 [12]	I						
Wagner et al., 2008 [7]						D*	
Uctasil et al., 2008 [4]			N				
Lohbauer et al., 2009 [8]	N						N
Saade et al., 2009 [23]					N		
El-korashy et al., 2010 [13]	I						I
Lucey et al., 2010 [34]				D	I		
Fróes-salgado et al., 2010 [16]	N	I	N				
Choudhary et al., 2011 [3]		I					
Nada et al., 2011 [9]					I		
dos Santos et al., 2011 [7]						D*	
Deb et al., 2011 [6]			N	D		N	I
Karaarslan et al., 2012 [5]						N	
Ayeb et al., 2014 [5]				D	I		
Calheiros et al., 2014 [21]	I						
Tauböck et al., 2015 [21]	I						
Yang et al., 2016 [22]						D	
Theobaldo et al., 2017 [14]	I				N		

## Conclusions

Preheating composite resins improves the degree of conversion, stiffness, marginal adaptability, and microhardness, according to the findings. Flexural strength is unbothered, polymerization shrinkage is hindered, and the microleakage results are unknown. However, more research with a bigger sample size and similar experimental settings is needed to establish the therapeutic importance of preheating.
